# Family Caregivers’ Experiences With Health Care Workers in the Care of Older Adults With Activity Limitations

**DOI:** 10.1001/jamanetworkopen.2019.19866

**Published:** 2020-01-24

**Authors:** Jennifer L. Wolff, Vicki A. Freedman, John F. Mulcahy, Judith D. Kasper

**Affiliations:** 1 Roger C. Lipitz Center for Integrated Health Care, Department of Health Policy and Management, Johns Hopkins Bloomberg School of Public Health, Baltimore, Maryland; 2 Institute for Social Research, University of Michigan, Ann Arbor

## Abstract

**Importance:**

Family and unpaid caregivers often play an active role in managing the care of older adults with activity limitations.

**Objective:**

To examine caregivers’ experiences with older adults’ health care workers.

**Design, Setting, and Participants:**

This survey study constitutes a secondary analysis of a sample of 1916 family and unpaid caregivers to 1203 community-living older adults with activity limitations who participated in the 2017 National Health and Aging Trends Study. Data analysis was performed January to August 2019.

**Exposures:**

Caregiver sociodemographic characteristics, caregiving intensity, and frequency speaking with or emailing older adults’ health care workers.

**Main Outcomes and Measures:**

Caregiver-reported experiences when interacting with older adults’ health care workers in the prior year, including being listened to, being asked about understanding of treatments, and being asked about help needed in managing older adults’ care.

**Results:**

Caregivers (mean [SE] age, 59.4 [0.5] years; 63.7% women) assisting community-living older adults with activity limitations reported that they never (56.3%), sometimes or rarely (33.0%), or often (10.7%) spoke with or emailed older adults’ health care workers in the prior year. Most caregivers who interacted with older adults’ health care workers reported being always (70.6%) or usually (18.2%) listened to and always (54.4%) or usually (17.7%) being asked about their understanding of older adults’ treatments. Fewer caregivers reported being always (21.3%) or usually (6.9%) asked whether they needed help managing older adults’ care, and nearly one-half (45.0%) were never asked. Caregivers who interacted with older adults’ health care workers often (vs sometimes or rarely) were more likely to report being always or usually listened to (94.8% vs 86.9%; *P* = .004), being asked about understanding treatments (80.1% vs 69.5%; *P* = .02), and being asked about needing help (40.8% vs 24.1%; *P* < .001). No other exposures were consistently associated with caregiver experiences. Measures of caregiving intensity, including caring for an older adult with dementia, were not associated with being listened to or asked about understanding, but were associated with being asked about needed help. Although caregivers of persons with dementia were more likely than caregivers of persons without dementia to report always being asked about needed help (26.9% vs 19.0%), a high percentage in both groups were never asked (41.2% vs 46.5%) (*P* = .007).

**Conclusions and Relevance:**

These findings reinforce the need for strategies to better support family and unpaid caregivers, who are the main source of assistance to older adults with physical and/or cognitive limitations.

## Key Points

**Question:**

What are family and unpaid caregivers’ experiences with health care workers in the care of older adults with activity limitations?

**Findings:**

In this national survey study, most caregivers reported that older adults’ health care workers always (70.6%) or usually (18.2%) listened to them and always (54.4%) or usually (17.7%) asked about their understanding of the older adult’s treatments, but fewer caregivers reported being always (21.3%) or usually (6.9%) asked whether they need help managing older adults’ care.

**Meaning:**

These findings reinforce the need for health system strategies to support family and unpaid caregivers, who are the main source of assistance to older adults with physical and/or cognitive limitations.

## Teaser

This survey study examines the experiences of family and unpaid caregivers for older adults when interacting with their health care workers, including being listened to, being asked about understanding of treatments, and being asked about needing help in managing care.

## Introduction

Family and other unpaid caregivers provide most of the assistance to community-living older adults with disability.^1^ In addition to helping with household and self-care activities, nearly one-half of caregivers assist with health care activities, such as managing medications, coordinating care, or attending medical encounters.^2^ Although caregivers often participate in the exchange of patients’ health information and in medical decision-making,^3,4,5^ supporting their involvement in health care interactions is not straightforward. Patients vary in their preferences for communication assistance,^6,7^ and caregivers vary in their knowledge of patient health conditions and priorities.^8,9^ Interacting with caregivers poses challenges for health care workers (eg, physicians, nurses, other clinicians, and social workers), who are responsible for ensuring patient privacy and promoting patient autonomy but are not reimbursed for additional time spent educating or counseling family and unpaid caregivers or nurturing productive partnerships.^10,11,12^

Available evidence—largely anecdotal and setting specific—has described interactions between family and unpaid caregivers and health care workers as being tense or adversarial.^11,13^ Although the communication challenges of surrogate decision-makers are well documented,^14,15,16,17^ little is known about the experiences of family and unpaid caregivers during routine interactions with older adults’ health care workers. Having a better understanding of caregivers’ experiences during interactions with older adults’ health care workers is especially timely given the growing interest in the contribution of social risk to outcomes of care.^18^ Recent changes to Medicare conditions of participation and reimbursement models that set forth an expectation and the possibility of more explicit caregiver support heighten the importance of better understanding critical elements of person-centered and family-centered care.^19,20^

This study draws on 2 linked nationally representative, population-based studies^21,22^ that are uniquely able to provide insight regarding the experiences of family and unpaid caregivers to a well-characterized sample of community-living older adults with physical or cognitive impairment. Our study has 2 broad objectives. First, we assess the frequency and nature of family and unpaid caregivers’ interactions with health care workers, including whether these interactions are associated with characteristics of older adults, family and unpaid caregivers, or caregiving circumstances and appraisal. In doing so, we examine caregivers’ perceptions of aspects of communication that have been identified as important in person-centered and family-centered care, including being listened to, being asked about understanding of treatments, and being asked about the need for help in managing older adults’ care.^23,24,25^ Second, we comparatively examine family caregivers’ experiences by whether they assist an older adult with or without dementia, recognizing that family caregivers’ interactions with health care workers are likely to be both more frequent and important in the context of impaired memory and judgment.^26,27^

## Methods

### Data

This study was approved by the institutional review board at the Johns Hopkins Bloomberg School of Public Health. This study follows the Strengthening the Reporting of Observational Studies in Epidemiology (STROBE) reporting guideline. Data for this study are drawn from the 2017 National Health and Aging Trends Study (NHATS)^21^ and the National Study of Caregiving (NSOC).^22^ The initial NHATS sample was drawn from the Medicare enrollment file in 2011 and replenished in 2015; written informed consent was obtained from participants.^21^ In 2017, the study was conducted with surviving beneficiaries aged 67 years and older. In-person interviews were conducted with NHATS study participants or with proxy respondents if the participant was unable to respond. Study participants were asked whether and how they performed daily activities in the month before the interview. Among those receiving assistance, a detailed roster was created that lists the relationship of each helper and the specific activities with which they provided help.

The NSOC^22^ is a nationally representative survey of family and other unpaid caregivers to older persons with activity limitations. Participants in the NSOC were eligible if they were family or other unpaid helpers to NHATS study participants receiving help with mobility, self-care, or household activities for health and functioning reasons or living in a residential care facility with supportive services. Upon obtaining oral consent, a telephone interview was conducted with up to 5 eligible helpers for each older adult. For older adults with more than 5 eligible helpers, 5 helpers were selected at random for interview.

Of 6312 older adults who participated in the 2017 NHATS, 2214 were included in the NSOC cross-sectional sampling frame, and 4676 of their helpers were eligible for the NSOC. Nonresponse to the NSOC can arise from the NHATS participant (who may refuse to provide contact information for helpers) or caregivers (who may refuse to participate). Participants in the NHATS did not provide contact information for 359 eligible family caregivers, and 1665 of the remaining 4317 eligible family caregivers could not be located or refused to respond. In total, 2652 family caregivers to 1697 older adults responded to the NSOC,^22^ yielding 92.4% and 61.9% first-stage and second-stage response rates, respectively. We excluded caregivers to 291 older adults who died and caregivers who were helping 323 older adults living in residential care or 101 adults living in nursing facilities because the nature of caregiving is likely to vary according to availability of services.

### Measures

Our main exposure measure is the frequency of caregivers’ interactions with older adults’ health care workers. Caregivers were asked to respond yes or no to the question “In the last year, did you ever speak to or email any of [older adult’s] medical providers about [his/her] care?” Those responding “no” were categorized as never interacting with older adults’ health care workers. Those responding “yes” were asked, “In the last year, how often did you speak to or email [older adult’s] medical providers about [his/her] care (often, sometimes, rarely)?” We grouped caregivers reporting they interacted with health care workers sometimes or rarely together and examined mutually exclusive categories of never, sometimes or rarely, and often.

Measures of caregiver-reported experiences when interacting with older adults’ health care workers were derived from 3 questions that were fielded to caregivers who reported interacting with older adults’ health care workers in the last year. These caregivers were told to think about the medical professional with whom they communicated with most often and were then asked, “In the last year, how often did that provider listen to what you had to say, ask if you understood [older adult’s] health treatments, and ask if you needed help managing [older adult’s] health treatments?” For these 3 dimensions of communication, we contrast caregivers responding “always” or “usually” with caregivers responding “sometimes” or “never.”

Additional measures included caregivers’ sociodemographic characteristics, caregiving intensity, and caregiving-related appraisal. Caregivers’ sociodemographic characteristics included age, gender, educational attainment, and relationship to the older adult (spouse, adult child, or other). Caregiving intensity included hours of care provided in the previous week, caregiving for 4 or more years, and types of assistance. We examined provision of help with types of assistance that have special relevance for interactions with health care workers, including medical tasks (tracking medications, administering injections, providing ostomy care or intravenous line care, and providing skin care, such as for wounds or sores) and health system logistics of care coordination.

We examined 2 measures of older adults’ physical and cognitive functioning, which have distinctive outcomes on the need for care: self-care and mobility limitations and dementia. We recognize that self-care and mobility limitations may impose the need for transportation or logistical assistance to access medical care, whereas the outcomes of dementia on memory, understanding, and reasoning may impose the need for help in the exchange of medical information and decision-making. We constructed a composite measure of self-care or mobility disability that reflects whether the older adult received help in the prior month with 1 or more self-care (eg, eating, dressing, bathing, and toileting) or mobility (eg, getting outside, getting around inside, and getting out of bed) activities. We used a composite measure of probable dementia constructed from self-reported physician diagnosis of Alzheimer disease or dementia, the AD8 dementia screening interview (administered to proxy respondents),^28^ and cognitive tests to evaluate memory, orientation, and executive function.^29^

Caregiving appraisal refers to a composite measure of caregiving strain (range, 0-9), constructed from 6 items that encompass appraised emotional, physical, and financial difficulty; having no time for oneself; being overwhelmed; and exhaustion. To assess caregiving-related difficulty, caregivers were first asked “Is helping emotionally/physically/financially difficult?” Those responding “yes” were then asked to rate the difficulty of helping in each domain on a scale from 1 (a little difficult) to 5 (very difficult). These items were scored as follows: no difficulty, 0 points; 1 to 3 (some difficulty), 1 point; and 4 to 5 (a lot of difficulty), 2 points. Affirmative responses to questions about having no time for oneself, being overwhelmed, and being exhausted were coded as 1, and negative responses were coded as 0. The composite measure has previously been found to have clinical relevance.^30,31^

### Statistical Analysis

We first assessed the frequency with which family and unpaid caregivers interacted with older adults’ health care workers in the prior year. We estimated the number and characteristics of family and unpaid caregivers reporting they never, sometimes or rarely, or often interacted with older adults’ health care workers. We then described family caregivers’ experiences during interactions with older adults’ health care workers. We compared family and unpaid caregiver sociodemographic characteristics and their caregiving intensity and appraisal by whether they reported that older adults’ health care workers always or usually listen to what they have to say, ask about understanding of older adults’ treatments, and ask about help needed by caregivers to manage older adults’ treatments, in comparison with those reporting that such interactions occurred sometimes or never. Finally, because family caregivers’ interactions with health care workers are particularly important in the context of impaired memory and judgment,^26,27^ and the care needs of persons with dementia are particularly demanding,^32^ we comparatively examined family caregivers’ experiences by whether they assist an older adult with or without dementia.

All analyses were conducted in SAS statistical software version 9.4 (SAS Institute) and Stata statistical software version 14 (StataCorp). Between-group differences were examined from *P* values associated with the Rao-Scott χ^2^ test for categorical measures and the adjusted Wald test for continuous measures, with 2-sided *P* < .05 considered statistically significant. We weighted our analytic sample to account for caregivers having different probabilities of selection and different probabilities of responding to the NSOC. To make accurate statements about the variance of those estimates, we applied design variables to account for NSOC’s complex sample design. Additional details about NSOC survey weights and sampling procedures have been published elsewhere.^33,34^ This secondary analysis was conducted between January and August 2019.

## Results

Our analytic sample included 1203 older adults living in traditional community settings in 2017 and their 1916 family and unpaid caregivers (mean [SE] age, 59.4 [0.5] years; 63.7% women). Among an estimated 17.4 million family and unpaid caregivers, more than one-half (56.3%) reported they did not interact with older adults’ health care workers in the prior year, whereas one-third (33.0%) reported speaking or emailing sometimes or rarely, and 10.7% reported speaking with or emailing older adults’ health care workers often (Table 1).

**Table 1.** Characteristics of Family and Unpaid Caregivers by Frequency of Interactions With Older Adults’ Health Care Workers^a^

Characteristic	Frequency of Interaction With Health Care Workers			*P* Value	All Caregivers
	Never	Sometimes or Rarely	Often		
Total caregivers, No. in millions (row %)^b^	9.8 (56.3)	5.7 (33.0)	1.9 (10.7)		17.4 (100.0)
Sociodemographic characteristics, No. (weighted column %)					
Age, mean (SE), y	59.2 (0.7)	60.4 (0.7)	57.5 (0.8)	.02	59.4 (0.5)
Female	616 (59.2)	463 (65.9)	216 (81.0)	<.001	1295 (63.7)
Beyond high school education	516 (53.0)	443 (69.6)	194 (74.2)	<.001	1153 (60.8)
Relationship to older adult					
Spouse	213 (21.3)	175 (30.4)	44 (16.8)	<.001	432 (23.8)
Daughter or son	402 (35.0)	332 (45.2)	177 (65.7)		911 (41.6)
Other	385 (43.7)	141 (24.4)	47 (17.5)		573 (34.5)
Caregiving intensity, No. (weighted %)					
Care, h/wk^c^					
0 to <10	555 (60.4)	296 (50.0)	70 (37.3)	<.001	921 (54.5)
10 to <20	176 (16.3)	121 (19.0)	52 (18.0)		349 (17.4)
≥20	269 (23.3)	231 (31.0)	146 (44.7)		646 (28.1)
Caregiving ≥4 y, No. (%)	619 (55.7)	452 (64.2)	198 (66.4)	.01	1269 (59.6)
Medical tasks, No. (%)					
Keep track of medications	413 (36.0)	420 (60.8)	226 (77.5)	<.001	1059 (48.6)
Skin care, eg, for wounds or sores	180 (15.3)	164 (26.2)	103 (37.1)	<.001	447 (21.2)
Ostomy care, intravenous care, testing blood	63 (6.1)	73 (11.1)	53 (17.1)	<.001	189 (8.9)
Administer injections	69 (7.2)	50 (7.4)	43 (13.4)	.05	162 (8.0)
Coordinate care	171 (14.8)	336 (49.1)	226 (84.6)	<.001	733 (33.6)
Assists older adult with dementia, No. (%)					
Yes	286 (22.5)	217 (25.9)	117 (36.6)	.001	620 (25.1)
No	714 (77.5)	431 (74.1)	151 (63.4)		1296 (74.9)
Assists older adults with self-care or mobility limitations, No. (%)					
Yes	576 (55.5)	409 (58.1)	177 (65.0)	.16	1162 (57.4)
No	424 (44.5)	239 (41.9)	91 (35.0)		754 (42.6)
Appraisal of caregiving, composite measure of caregiving strain, No. (%)^d^					
Little or none	801 (79.3)	435 (69.0)	137 (51.3)	<.001	1373 (69.0)
Moderate or high	199 (20.7)	213 (31.0)	131 (48.7)		543 (27.1)

^a^ Data are from the 2017 National Health and Aging Trends Study^21^ and 2017 National Study of Caregiving.^22^

^b^ In the unweighted sample, 1000 caregivers reported never, 648 reported sometimes or rarely, and 268 reported often. Estimates are weighted.

^c^ Caregivers with missing hours (92) were imputed using multivariate imputation including caregiver gender, relationship to the older adult, older adult’s dementia status, and provision of self-care help using fully conditional selection by days helped per week.

^d^ Composite measure of strain from 6 items that assess physical, emotional, and financial difficulty, exhaustion, role overload, and lack of time for oneself due to caregiving (range, 0-9), with a score of 0 or 1 denoting little or none (1373 participants) and 2 to 9 denoting some or high strain  (543 participants).

Compared with family and unpaid caregivers who interacted with health care workers sometimes or rarely or never, those who interacted with older adults’ health care workers often were younger (mean [SE] age, 57.5 [0.8] years vs 59.2 [0.7] years for never and 60.4 [0.7] years for sometimes or rarely; *P* = .02) and more likely to be female (81.0% vs 59.2% for never and 65.9% for sometimes or rarely; *P* < .001), better educated (education beyond high school, 74.2% vs 53.0% for never and 69.6% for sometimes or rarely; *P* < .001), an adult child (65.7% vs 35.0% for never and 45.2% for sometimes or rarely; *P* < .001), and to have been providing care for 4 or more years (66.4% vs 55.7% for never and 64.2% for sometimes or rarely; *P* = .01). Caregivers who interacted with health care workers often were providing care of greater intensity as measured by providing care 20 or more hours per week (44.7% vs 31.0% for those interacting sometimes or rarely and 23.3% for those with no contact; *P* < .001) and to be assisting with medically oriented tasks and care coordination. Caregivers who interacted with health care workers often were more likely to be caring for an older adult with dementia (36.6% of those interacting often vs 25.9% and 22.5% interacting sometimes or rarely and never, respectively; *P* = .001) and to report moderate or high caregiving-related strain (48.7% of those interacting often compared with those interacting with older adults’ health care workers less frequently vs 31.0% and 20.7% interacting sometimes or rarely and never, respectively; *P* < .001).

**Figure 1.**
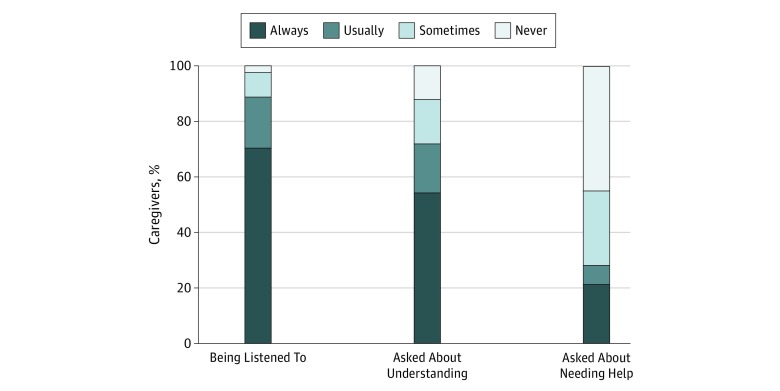
Family and Unpaid Caregivers’ Experiences With Older Adults’ Health Care Workers Estimates are weighted and reflect the experiences of family and unpaid caregivers of being listened to, being asked about understanding of treatments, and being asked about needing help always, usually, sometimes, or never. Data are from an unweighted sample of family and unpaid caregivers who interacted with older adults’ health care workers within the prior year and who reported on their experiences of being listened to (913 participants), being asked about understanding (912 participants), and being asked about needing help in managing older adults’ treatments (906 participants).

Family and unpaid caregivers’ experiences with older adults’ health care workers varied markedly by type of communication support (Figure 1). Most caregivers reported that older adults’ health care workers always (70.6%) or usually (18.2%) listened to what they had to say (total, 88.8%) and always (54.4%) or usually (17.7%) asked about their understanding of older adults’ health treatments (total, 72.1%). Fewer caregivers reported that older adults’ health care workers always (21.3%) or usually (6.9%) asked about needed help managing older adults’ treatments (total, 28.2%). Approximately 1 in 4 (26.9%) reported they were sometimes asked, and nearly one-half (45.0%) were never asked.

Caregiver sociodemographic characteristics and caregiving-associated appraisal were not associated with being listened to, asked about understanding, or asked about the need for help in managing care during interactions with older adults’ health care workers (Table 2). Those family and unpaid caregivers who interacted with older adults’ health care workers often (vs sometimes or rarely) were more likely to report being listened to (94.8% vs 86.9%; *P* = .004), being asked about understanding treatments (80.1% vs 69.5%; *P* = .02), and being asked about needing help (40.8% vs 24.1%; *P* < .001). No other characteristics were consistently associated with all 3 types of communication support. Measures of caregiving intensity were not associated with caregivers’ experiences of being listened to or asked about understanding but were associated with being asked about needed help. Those providing more hours of care per week (35.4% of those helping ≥20 hours vs 25.6% and 24.0% of those helping 10 to <20 and 0 to <10 hours, respectively; *P* = .01) and helping with medically oriented tasks, such as keeping track of medications (34.4% vs 16.7%; *P* < .001), administering injections (42.4% vs 26.8%; *P* = .02), and helping with ostomy or intravenous line care (40.7% vs 26.4%; *P* = .02), were more likely to report being always or usually asked about needed help. Caregivers assisting an older adult with dementia (37.3% vs 24.5%; *P* < .001) or self-care or mobility limitations (32.0% vs 22.5%; *P* = .02) were more likely than caregivers assisting persons without dementia or mobility limitations to report being always or usually asked about needing help.

**Table 2.** Characteristics of Family and Other Unpaid Caregivers Who Interacted With Older Adults’ Health Care Workers, Stratified by Experiences With Care^a^

Characteristics	Health Care Professionals Listen to What You Have to Say			Health Care Professionals Ask About Your Understanding of Treatments			Health Care Professionals Ask About Needing Help		
	Always or Usually	Sometimes or Never	*P* Value	Always or Usually	Sometimes or Never	*P* Value	Always or Usually	Sometimes or Never	*P* Value
Total caregivers, No. in millions (row %)^b^	6.7 (88.8)	0.8 (11.2)		5.5 (72.1)	2.1 (27.9)		2.1 (28.2)	5.4 (71.8)	
Sociodemographic characteristics									
Age, mean (SE), y	59.6 (0.6)	60.3 (2.3)	.76	59.0 (0.7)	61.3 (1.1)	.10	58.0 (0.9)	60.2 (0.7)	.06
Gender, No. (row %)									
Female	615 (90.3)	62 (9.7)	.13	499 (72.1)	177 (27.9)	.99	215 (27.4)	456 (72.6)	.61
Male	201 (85.5)	35 (14.5)		170 (72.1)	66 (27.9)		64 (30.0)	171 (70.0)	
Educational attainment, No. (row %)									
High school or less	246 (86.9)	31 (13.1)	.32	217 (73.2)	62 (26.8)	.75	80 (27.6)	195 (72.4)	.86
Beyond high school	570 (89.6)	66 (10.4)		452 (71.6)	181 (28.4)		199 (28.4)	432 (71.6)	
Relationship to older adult, No. (row %)									
Spouse	192 (86.7)	25 (13.3)	.12	145 (66.6)	73 (33.4)	.25	56 (25.3)	159 (74.7)	.69
Daughter or son	465 (91.5)	43 (8.5)		387 (75.4)	119 (24.6)		161 (29.8)	342 (70.2)	
Other	159 (85.3)	29 (14.7)		137 (71.4)	51 (28.6)		62 (28.1)	116 (71.9)	
Caregiving intensity									
Care, h/wk, No. (%)^c^									
0 to <10	320 (88.1)	46 (11.9)	.36	249 (68.4)	117 (31.6)	.11	92 (24.0)	271 (76.0)	.01
10 to <20	154 (86.0)	19 (14.0)		124 (71.5)	49 (28.5)		50 (25.6)	123 (74.4)	
≥20	342 (91.3)	32 (8.7)		296 (77.4)	77 (22.6)		137 (35.4)	233 (64.6)	
Duration of caregiving, y, No. (row %)									
<4	235 (89.2)	31 (10.8)	.83	195 (76.5)	69 (23.5)	.09	83 (28.9)	180 (71.1)	.80
≥4	581 (88.6)	66 (11.4)		474 (69.7)	174 (30.3)		196 (27.8)	447 (72.2)	
Medical tasks									
Keeps track of medications, No. (row %)									
Yes	583 (90.6)	60 (9.4)	.07	490 (75.3)	154 (24.7)	.05	229 (34.4)	409 (65.6)	<.001
No	233 (85.5)	37 (14.5)		179 (66.1)	89 (33.9)		50 (16.7)	218 (83.3)	
Skin care, eg, for wounds or sores, No. (row %)									
Yes	236 (90.4)	30 (9.6)	.36	188 (69.4)	77 (30.6)	.42	95 (31.6)	168 (68.4)	.17
No	580 (88.2)	67 (11.8)		481 (73.2)	166 (26.8)		184 (26.8)	459 (73.2)	
Ostomy care, intravenous care, testing blood, No. (row %)									
Yes	115 (93.4)	9 (6.6)	.12	98 (76.6)	27 (23.4)	.38	58 (40.7)	66 (59.3)	.02
No	701 (88.2)	88 (11.8)		571 (71.4)	216 (28.6)		221 (26.4)	561 (73.6)	
Administer injections, No. (row %)									
Yes	84 (91.6)	8 (8.4)	.48	78 (79.2)	15 (20.8)	.28	44 (42.4)	45 (57.6)	.02
No	732 (88.5)	89 (11.5)		591 (71.4)	228 (28.6)		235 (26.8)	580 (73.2)	
Coordinate care, No. (row %)									
Yes	513 (91.9)	47 (8.1)	.01	426 (74.7)	135 (25.3)	.16	189 (31.1)	367 (68.9)	.08
No	303 (84.7)	50 (15.3)		243 (68.4)	108 (31.6)		90 (24.2)	260 (75.8)	
Frequency of interactions, No. (row %)									
Sometimes or rarely	562 (86.9)	83 (13.1)	.004	447 (69.5)	198 (30.5)	.02	167 (24.1)	471 (75.9)	<.001
Often	254 (94.8)	14 (5.2)		222 (80.1)	45 (19.9)		112 (40.8)	156 (59.2)	
Assists older adult with dementia, No. (row %)									
Yes	306 (89.4)	26 (10.6)	.83	260 (77.7)	71 (22.3)	.08	135 (37.3)	193 (62.7)	<.001
No	510 (88.6)	71 (11.4)		409 (69.8)	172 (30.2)		144 (24.5)	434 (75.5)	
Assists older adults with self-care or mobility limitation, No. (row %)									
Yes	523 (90.1)	61 (9.9)	.37	426 (74.6)	157 (25.4)	.21	192 (32.0)	387 (68.0)	.02
No	293 (86.9)	36 (13.1)		243 (68.4)	86 (31.6)		87 (22.5)	240 (77.5)	
Appraisal of caregiving, composite measure of strain, No. (%)^d^									
Little or none	512 (90.2)	58 (9.8)	.19	435 (73.9)	144 (26.1)	.24	163 (27.8)	403 (72.2)	.82
Moderate or high	304 (86.2)	39 (13.8)		244 (68.8)	99 (31.2)		116 (28.9)	224 (71.1)	

^a^ Data are from the 2017 National Health and Aging Trends Study^21^ and 2017 National Study of Caregiving.^22^

^b^ In the unweighted sample, 816 caregivers reported that health care workers always or usually and 97 reported that health care workers sometimes or never listen to what they have to say, 669 caregivers reported they were always or usually and 243 reported they were sometimes or never asked about understanding of treatment, and 279 reported they were always or usually and 627 reported they were sometimes or never asked about needing help. Estimates are weighted.

^c^ Caregivers with missing hours (92) were imputed using multivariate imputation including caregiver gender, relationship to the older adult, older adult dementia status, and provision of self-care help using fully conditional selection by days helped per week.

^d^ Composite measure of strain from 6 items that assess physical, emotional, and financial difficulty, exhaustion, role overload, and lack of time for oneself due to caregiving (range, 0-9), with a score of 0 or 1 denoting little or none (570 participants) and a score of 2 to 9 denoting some or high strain (343 participants).

**Figure 2.**
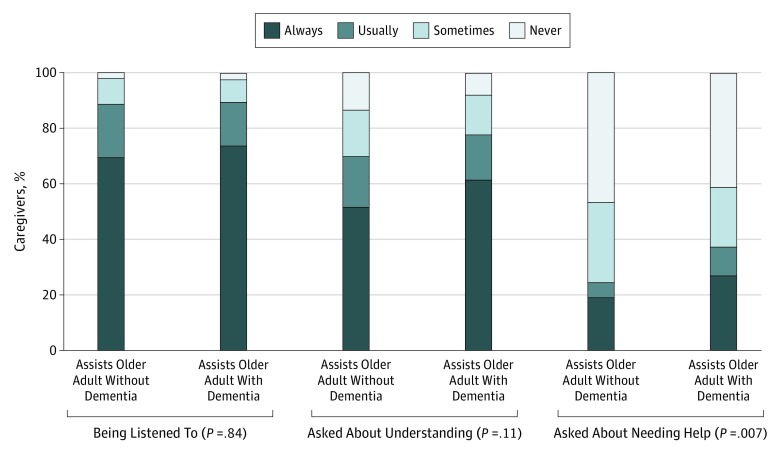
Family and Unpaid Caregivers’ Experiences With Older Adults’ Health Care Workers, Stratified by Dementia Status Estimates are weighted and reflect the experiences of family and unpaid caregivers to older adults without and with dementia of being listened to, being asked about understanding of treatments, and being asked about needing help always, usually, sometimes, or never. Data are from an unweighted sample of family and unpaid caregivers who interacted with older adults’ health care workers within the prior year and who reported on their experiences of being listened to (581 caregivers for patients without dementia, 332 caregivers for patients with dementia), being asked about understanding (581 caregivers for patients without dementia, 331 caregivers for patients with dementia), and being asked about needing help managing older adults’ treatments (578 caregivers for patients without dementia, 328 caregivers for patients with dementia).

We compared the experiences of caregivers for persons with and without dementia during interactions with health care workers (Figure 2). Caregivers for persons with dementia were no more likely than caregivers for persons without dementia to report being listened to and being asked about understanding of treatments by the older adults’ health care workers, but were more likely to report they were always asked about needed help managing older adults’ treatments (26.9% vs 19.0%) and were less likely to report being never asked (41.2% vs 46.5%) (*P* = .007).

## Discussion

To our knowledge, this study provides the first national information about the experiences of family and unpaid caregivers during routine interactions with older adults’ health care workers. We found that caregivers overwhelmingly reported positive elements of communication when interacting with health care workers about older adults’ health and treatment but were less often asked about their need for help in managing older adults’ care. Although our results depict a generally respectful and constructive family-clinician partnership, they raise important questions about the role of health care workers in supporting the needs of family caregivers, who are so critical in the context of serious illness and late-life disability.

Professional societies,^10,35^ advocacy organizations,^24,36^ and consensus committees^1,37^ among others^38,39^ have called for routine assessment of family caregivers as an element of high-quality clinical care and robust systems of long-term services and supports. Our study portrays a mixed state of affairs: although nearly one-third (28.2%) of caregivers reported being always or usually asked by health care workers about whether they needed help in managing older adults’ treatments, nearly one-half (45.0%) were never asked. Our finding that caregivers providing more hours of care to older adults in worse health were more likely to be asked about needed help suggests that health care workers generally recognized that higher-intensity caregivers may especially need additional support. Although consensus guidelines regarding the circumstances under which an assessment should be performed do not now exist,^1,40^ our finding that more than 4 in 10 caregivers for persons with dementia were not asked about needed help suggests room for improvement.

Nearly 9 in 10 family caregivers (88.8%) reported that older adults’ health care workers always or usually listened to what they had to say and nearly 3 in 4 (72.1%) reported being always or usually asked about their understanding of older adults’ medical treatments. The favorable communication reported by family caregivers when interacting with older adults’ health care workers stands in stark contrast with the challenges that have been widely described by family caregivers when reporting on their experiences navigating health system demands.^11,38,41^ There are several explanations for the disconnect between our findings and the prevailing literature. First, prior studies have been largely anecdotal^42,43^ or specific to acute-care settings^14,16,44^ and may, therefore, not generalize to the experiences of family caregivers in routine care. Second, the challenges that have been reported may be less due to interpersonal interactions with individual health care workers than systemwide deficiencies, such as fragmented care,^45,46^ barriers to appropriate information access about the patient’s health or treatments,^47,48^ or issues specific to surrogate decision-making, such as knowledge gaps relating to patient treatment goals and wishes.^17,49^ Third, our study focuses on the experiences of caregivers who spoke or emailed with health care workers in the prior year and excludes perspectives of the more than half of caregivers who did not interact with health care professionals, some of whom may have experienced difficulties accessing care or who may purposefully avoid seeking out older adults’ health care workers because of prior experiences.

Given the important consequences of interpersonal communication between patients and clinicians in the delivery of high-quality care, appropriate services use, and health outcomes,^50^ it is encouraging that elements of information exchange were rated especially highly in the subgroup of caregivers with particularly demanding responsibilities who interacted with older adults’ health care professionals most often. Although family caregivers commonly accompany older adults to medical encounters,^3,4^ actively participate in face-to-face discussion,^3^ and are influential in adherence and care coordination,^1,3,51^ surprisingly little is known about the frequency, nature, and consequences of these interactions. There is a growing appreciation that understanding what people view to be important is foundational to the measurement and delivery of person-centered and family-centered care.^23^ Findings from this study and others^7,25^ suggest a benefit to assessing family perspectives in the measurement of care quality. Because family caregivers are often heavily involved in the care of persons with diminished capacity to self-report on experiences with care,^2,32^ incorporating family caregiver-reported information may be especially important to ensuring that assessments of care quality incorporate all perspectives. Newly developing methods and instruments are encouraging in this regard.^25,52^

### Strengths and Limitations

Several strengths and limitations of our study merit comment. Strengths of our study include drawing on nationally representative population-based data for a well-characterized sample of older adults and their family and unpaid caregivers. Although our study appropriately draws on caregiver-reported measures when reflecting on their experiences of care, self-reported information is subject to potential misclassification for measures such as the frequency of interactions. As a secondary analysis of a national survey, our study was constrained to available information regarding interactions with medical professionals, and we are unable to comment on factors such as the type or specialty of health care workers, their gender, their years in practice, the duration of their relationship with the older adult, and their communication style, all of which are known to be associated with interpersonal rapport and trust.^50,53^ We are unable to differentiate caregivers’ expectations or preferences for actively engaging with health care workers, or how patient expectations or preferences about the involvement of their caregiver are associated with these interactions. Although we examined specific elements of interpersonal communication, as opposed to a psychometrically validated multi-item instrument, the measures we assess are directly pertinent to aspects of care that have been identified as important to patients and families,^25,54^ as well as clinicians and policy makers who seek to improve care for those with chronic and disabling conditions.^39^

## Conclusions

Well-coordinated, team-based care is an important element in the delivery of care that is safe, efficient, and high quality.^55^ Our study affords a novel perspective on such care by contributing insight regarding the experiences of family and unpaid caregivers when interacting with older adults’ health care workers. The high percentage of caregivers who reported being well supported when communicating with health care workers about older adults is indicative of their visible and better recognized role in maintaining the health and well-being of older adults with physical and/or cognitive limitations; that caregivers were less likely to report being asked about needed help reflects the greater ambiguity in clinician responsibility toward supporting family caregivers’ needs. These findings are directly relevant to the movement to recognize family as contributing members of an interdisciplinary care team^35,56^ and contribute benchmark information that may helpful in guiding and monitoring progress toward the advancement of person-centered and family-centered care.^23^
